# Sigmoid Colon Cancer Masquerading as a Right Incarcerated Inguinal Hernia: A Case Study and Literature Review

**DOI:** 10.3389/fsurg.2022.832771

**Published:** 2022-02-17

**Authors:** Jianfeng Zhang, Yujie Tang, Xueliang Wu, Guiying Wang, Tian Li

**Affiliations:** ^1^The Second Department of General Surgery, The Fourth Hospital of Hebei Medical University, Shijiazhuang, China; ^2^School of Basic Medicine, Hebei Medical University, Shijiazhuang, China; ^3^Department of General Surgery, The First Affiliated Hospital of Hebei North University, Shijiazhuang, China; ^4^Gastrointestinal Surgery Department, The Third Hospital of Hebei Medical University, Shijiazhuang, China; ^5^School of Basic Medicine, The Fourth Military Medical University, Xi'an, China

**Keywords:** sigmoid colon cancer, incarcerated hernia, physical examination, hernia repair, colectomy

## Abstract

**Background:**

Indirect inguinal hernia and sigmoid colon cancer are both common diseases, but carcinoma within the hernia sac is rare. We present a case of sigmoid colon cancer masquerading as a right incarcerated inguinal hernia. Since such a presentation is rare, and the correct diagnosis is usually made intraoperatively, there is still no consensus on the best treatment modality for such patients.

**Case Presentation:**

A 70-year-old man presented to our hospital on September 20, 2020, with a right inguinal mass that had been painful for half a month, accompanied by symptoms of difficult defecation. The bulge was originally found at least 60 years before admission. There was no pain at the time; however, the mass enlarged progressively during the last 3 years. The right scrotum and groin area were obviously enlarged (~20 × 20 cm) and tender. Inside the scrotum, a circumscribed medium-hard mass (diameter 5 cm) that was palpable, with ill-defined borders and translational mobility was detected. The computed tomography (CT) scan showed a right blood vessel-containing strangulated inguinal hernia; the sigmoid colon showed focal wall thickening as it was in proximity to the inguinal hernia. Based on the biopsy results, a pathologic diagnosis of high-grade intraepithelial neoplasia was made. The preliminary diagnosis was that of sigmoid carcinoma and right incarcerated inguinal hernia. Emergency laparoscopic exploration, open sigmoid radical resection andright inguinal hernia repair were performed under general anesthesia. The patient recovered successfully and was discharged 1 week after the operation. One month after surgery, no discomfort and signs of recurrence were found.

**Conclusions:**

The combination of colorectal cancer and inguinal hernia is uncommon, and detailed preoperative physical examination and imaging studies may contribute to the establishment of a correct diagnosis. The selection of appropriate surgical methods ensures good therapeutic results.

## Background

Indirect inguinal hernia and sigmoid colon cancer are both common diseases, but carcinoma within the hernia sac is rare. We present a case of sigmoid colon cancer masquerading as a right incarcerated inguinal hernia. Since such a presentation is rare, and the correct diagnosis is usually made intraoperatively, there is still no consensus on the best treatment modality for such patients.

## Case Presentation

### Chief Complaints

A 70-year-old man presented to our hospital on September 20, 2020, with a right inguinal mass that had been painful for half a month, accompanied by symptoms of difficult defecation.

### History of Present Illness

The bulge was originally found at least 60 years before admission. There was no pain at the time; however, the mass enlarged progressively during the last 3 years.

### History of Past Illness

The patient had a free previous medical history.

### Personal and Family History

There was no pertinent personal or family history of illness.

### Physical Examination

**Figure 1:**
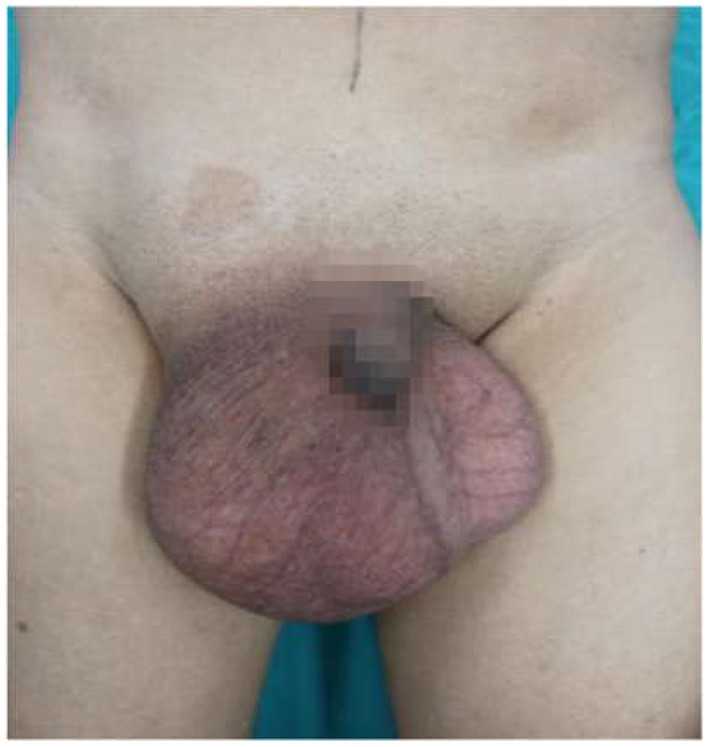
Appearance of the inguinoscrotal hernia.

The right scrotum and groin area were obviously enlarged (~20 × 20 cm) and tender. Inside the scrotum, a circumscribed medium-hard mass (diameter 5 cm) that was palpable, with ill-defined borders and translational mobility was detected. The mass could not be returned to the abdominal cavity in the supine position, and the transillumination test of the scrotum was negative (Figure 1).

### Imaging Examinations

**Figure 2:**
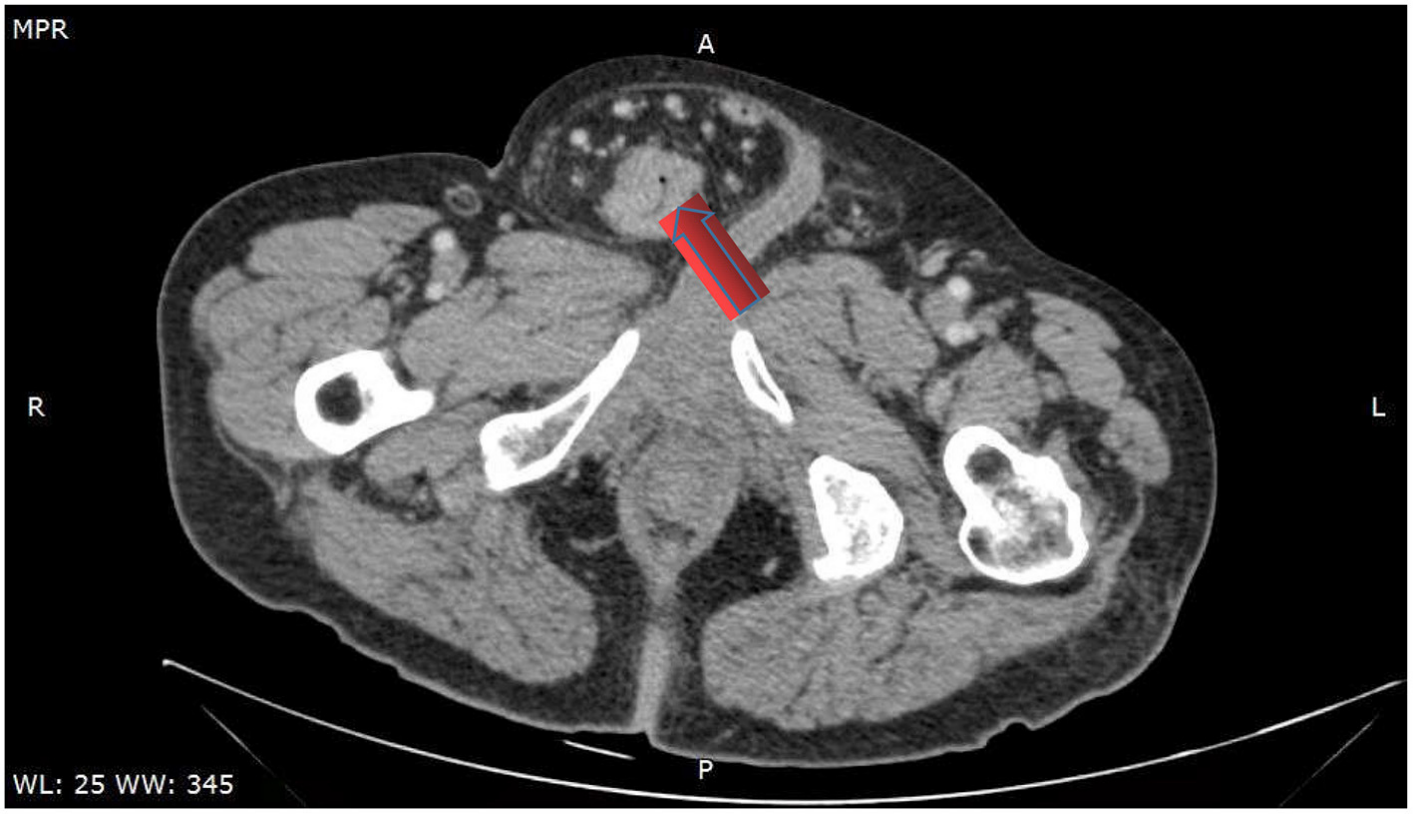
Computed tomography scan showing localized wall thickness of the sigmoid colon in the right groin.

**Figure 3:**
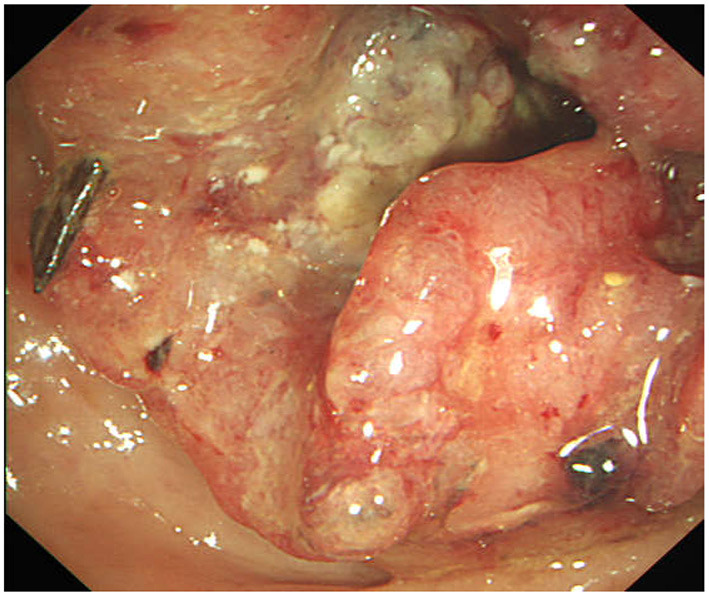
Endoscopic view, showing the sigmoid colon almost completely obstructed by the tumor.

The abdominal CT scan showed a right blood vessel-containing strangulated inguinal hernia; the sigmoid colon showed focal wall thickening as it was in proximity to the inguinal hernia (Figure 2). Chest CT shows emphysema in the right lung, nothing else unusual. The enhancement scan showed that the inhomogeneous enhancing mass had an irregular margin and unclear boundaries with surrounding tissues. Colonoscopy showed an annular stricture of the sigmoid colon caused by a tumor located 18–25 cm from the anal verge, indicative of malignancy (Figure 3).

### Final Diagnosis

Based on the imaging results, the mass was diagnosed as sigmoid colon wall thickening, consistent with cancer and right inguinal hernia. Based on the biopsy results, a pathologic diagnosis of high-grade intraepithelial neoplasia was made. The preliminary diagnosis was that of sigmoid carcinoma and right incarcerated inguinal hernia.

### Treatment

**Figure 4:**
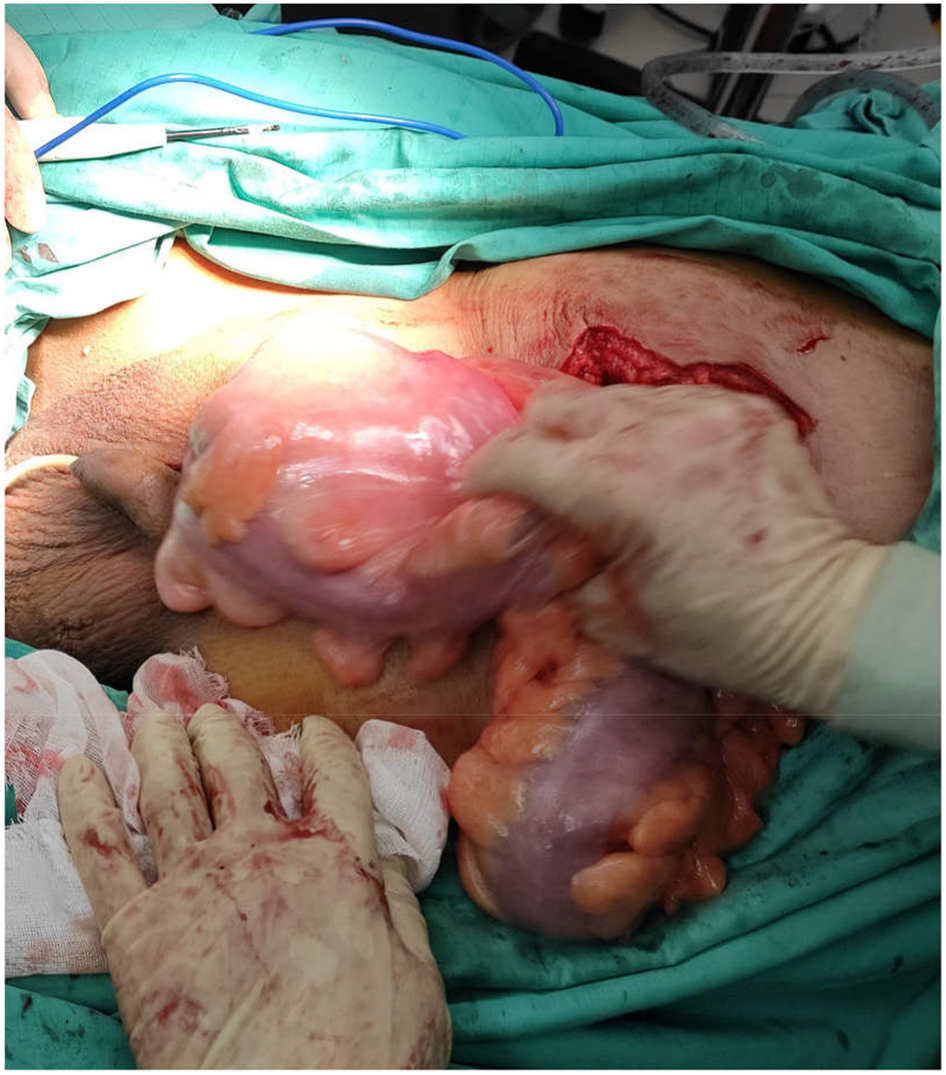
Surgical views showing segmental wall thickness of the sigmoid colon within the hernia sac.

Emergency laparoscopic exploration, open sigmoid radical resection and right inguinal hernia repair were performed under general anesthesia on October 29, 2020. No obvious metastasis was found on laparoscopic exploration. The sigmoid colon and its mesentery herniated into the right scrotum through the right inguinal ring; the inner ring was thickened, ~4 cm in diameter. The saccular contents could not be returned to the abdominal cavity. Hence, we converted to open surgery. The saccular contents could not be pulled out of the hernia ring through the median abdominal incision. Therefore, an inguinal incision was added. A huge hernia sac was identified upon incision. Upon opening the sac, we observed that a 5 × 5 cm hard mass of the sigmoid colon was tightly adhered to the sac; the incarcerated sigmoid colon was about 35 cm long and the serosa had been invaded; enlarged lymph nodes were visible in the mesentery (Figure 4). After the incision and enlargement of the inner ring, the sigmoid colon was completely returned to the abdominal cavity. Intraoperative colonoscopy showed no proximal bowel lesion. Subsequently, radical resection of the sigmoid colon and end-to-end anastomosis of the descending colon and rectum were performed. Meanwhile, the hernia was repaired by primary closure (Bassini technique) without using a mesh on account of possible contamination of the operative field during surgery.

### Outcome and Follow-Up

The patient recovered successfully and was discharged 1 week after the operation. 16 month after surgery, no discomfort and signs of recurrence were found. Findings of the physical examination and related auxiliary examinations were unremarkable.

## Discussion and Conclusions

Inguinal hernia is the most common external abdominal hernia, occurring in 1–5% of the population. Twenty million inguinal hernia repairs are completed annually worldwide (1). The combination of colorectal cancer and inguinal hernia is very rare. In 1938, Gerhardt first reported a case of colon cancer incarcerated in a hernia entering the groin, after which several similar cases were reported (2). Such cases accounted for ~0.03–0.5% among all inguinal hernias (3, 4). A current review of the literature on inguinal hernias containing colon carcinoma revealed that 16 cases were reported between 2003 and 2020 (including this case) (Table 1). In all these cases, the patients were men aged between 44 and 88 years (median, 71.5 years). Most of the hernias were located on the left side (75.0%). In the majority of the cases, the hernias contained sigmoid colon cancer (82.5%), while the others contained the descending colon and the ascending colon; in most cases, the contents were incarcerated (82.5%). In only half the cases (50.0%), the condition was correctly diagnosed before surgery, while in the rest of the cases, the issue was detected during an emergency operation. The case reported by the authors was unique in that the patient had a long history (over 60 years) of inguinal hernia. Herniation of sigmoid carcinoma into the right scrotum is relatively rare and was diagnosed preoperatively. No association between inguinal hernia and colonic cancer (5, 6). We believe that the long length of sigmoid colon and mesentery creates favorable conditions for the tumor herniation into the scrotum, gradually developing from reversible hernia to irreducible hernia, culminating to an incarcerated hernia. Most of the patients underwent open surgery (82.2%) and combined (abdominal and inguinal) incisions were made (62.5%). For the colon, the Hartmann procedure was performed in most cases (56.3%); for the inguinal hernia, primary closure (58.3%) was carried out. However, two patients (16.7%) underwent repair with interrupted stitches and absorbable mesh reinforcement (7, 10). The length of hospital stay after surgery was 7–35 days (median, 10 days); one patient died on the fifth day after the operation (11). Postoperative pathology showed that all tumors were in the T3 or T4 stage.

**Table 1:** Reported cases of colon cancer in an inguinal hernia sac between 2003 and 2020.

**References**	**Year**	**Sex**	**Age**	**Hernia side**	**Tumor location**	**Incarcerated or not**	**Correct diagnosis before operation**	**Surgical approach**	**Open/** **laparoscopy**	**Perforated or not**	**Operation for colon**	**Operation for hernia**	**T stage**	**Hospital time (days)**
Kouraklis et al. (7)	2003	Male	79	Left	Sigmoid colon	Incarcerated	No	Inguinal	Open	Yes	Sigmoidectomy	Not done, absorbable mesh during second operation	Not mentioned	10
Boormans et al. (8)	2006	Male	44	Right	Sigmoid colon	Incarcerated	No	Combine	Open	Yes	Sigmoidectomy (Hartmann procedure)	Not mentioned	T4	35
Slater et al. (6)	2008	Male	73	Left	Sigmoid colon	Incarcerated	No	Combine	Open	Yes	Sigmoidectomy (Hartmann procedure)	High ligation	T4	Not mentioned
Slater et al. (6)	2008	Male	66	Left	Sigmoid colon	Incarcerated	No	Combine	Open	No	Sigmoidectomy (Hartmann procedure)	High ligation	T3	Not mentioned
Sakorafas and Peros (9)	2008	Male	85	Right	Sigmoid colon	Incarcerated	No	Combine	Open	Yes	Sigmoidectomy (Hartmann procedure)	Bassini	Not mentioned	15
Ruiz-Tovar et al. (10)	2009	Male	67	Left	Sigmoid colon	Incarcerated	Yes	Combine	Open	Yes	Sigmoidectomy	Lichtenstein	T3	7
Ko et al. (11)	2010	Male	84	Left	Sigmoid colon	Incarcerated	Yes	Abdominal	Open	Yes	Sigmoidectomy (Hartmann procedure)	Not mentioned	T3	5 (expired)
Tan et al. (12)	2013	Male	63	Left	Sigmoid colon	Incarcerated	Yes	Combine	Open	Yes	Sigmoidectomy (Hartmann procedure)	Primary sutures	T4	10
Kanemura et al. (13)	2014	Male	67	Left	Sigmoid colon	non-Incarcerated	Yes	Abdominal	laparoscopy	No	Sigmoidectomy	High ligation	T3	12
Kulasegaran et al. (14)	2016	Male	66	Left	Sigmoid colon	Incarcerated	No	Combine	Open	Yes	Sigmoidectomy (Hartmann procedure)	Not mentioned	T3	Not mentioned
Diao and Ghosh (15)	2016	Male	48	Left	Transverse colon	Incarcerated	No	Inguinal	Open	Yes	Transverse colectomy	Primary closure	T3	8
Chern et al. (2)	2018	Male	83	Right	Ascending colon	Non-incarcerated	Yes	Abdominal	laparoscopy	No	Right hemicolectomy	Primary closure	T4	7
Mizuno et al. (3)	2019	Male	73	Left	Sigmoid colon	Incarcerated	Yes	Combine	Open	Yes	Sigmoidectomy (Hartmann procedure)	Marcy	T3	29
Baldi (4)	2020	Male	88	Left	Sigmoid colon	Incarcerated	Yes	Combine	Open	No	Sigmoidectomy	Not mentioned	T3	Not mentioned
Sabra et al. (1)	2020	Male	87	Left	Sigmoid colon	Incarcerated	No	Inguinal	Open	Yes	Sigmoidectomy (Hartmann procedure)	Primary closure	T4	A few days
This case (NA)	2020	Male	70	Right	Sigmoid colon	Incarcerated	Yes	Combine	Laparoscopy and open	No	Sigmoidectomy	Bassini	T3	7

In clinical practice, the main symptom that patients present with is pain or mass in the inguinal region, which is common in the case of inguinal hernia. Therefore, there is a possibility of misdiagnosis if detailed physical examination, imaging, and laboratory tests are not conducted. Because of the anatomical characteristics of the long and tortuous sigmoid colon, hernias may be more common on the left side. In the cases reviewed here, correct diagnosis before surgery was possible in all patients who were examined by ultrasonography, CT, or endoscopy. Takashi et al. pointed out that enhanced CT can significantly improve the accuracy of preoperative diagnosis and avoid the need for taking inappropriate treatment decisions (3, 4). Hironori et al. believed that intestinal blood supply disorders are more likely to occur because of the existence of tumors, which has a significant impact on disease prognosis; for instance, patients may have to undergo operations two or more time (3). In our case, the CT images revealed the thickening of the sigmoid colon wall, which was then confirmed through colonoscopy and pathology examinations. Surgery is the first-line of treatment after diagnosis. Chern et al. considered that for reducible hernias, the colon and its mesentery should be returned to the abdominal cavity through a laparoscopic procedure, then radical resection of the colon cancer must be carried out, followed by hernia repair (2, 13). Open surgery should be performed in cases of strangulated hernia. The surgical approach can be transabdominal, inguinal, or both, depending on the patient's specific conditions, intraoperative exploration, and the surgeon's experience (7, 11). Combined approaches were used in this case. Regardless of the approach, it is important to achieve a resection based on sound oncological principles and ensure a secure hernia repair. Inguinal hernia repair requires a clean wound, while sigmoid colon cancer excision is a clean-contaminated operation or even a contaminated operation when there is a perforated malignant tumor. In principle, a biological patch should not be used in such procedures for hernia repair. Simple suture repair is possible, such as in Furgeson, Bassini, and Halsted methods, which should be selected according to the specific condition of patients to avoid the occurrence of postoperative infection (6, 10). However, Jaime et al. believed that a biological patch can be used to reduce postoperative pain and hernia recurrence while carefully exploring the bowel without perforation and abdominal cavity contamination (10). However, in most of the cases, there was tumor perforation, and the postoperative pathology indicated late tumor stages (T3 or T4). Therefore, R0 resection is particularly important to reduce tumor recurrence, and combined incisions are more convenient for surgery (1, 2). The Hartmann procedure is preferred in order to avoid complications such as anastomotic leakage and abdominal infection because most patients exhibit intestinal obstruction, perforation, and other high-risk conditions (15).

The combination of colorectal cancer and inguinal hernia is uncommon, and detailed preoperative physical examination and imaging studies may contribute to the establishment of a correct diagnosis. The selection of appropriate surgical methods ensures good therapeutic results.

## Data Availability Statement

The original contributions presented in the study are included in the article/supplementary material, further inquiries can be directed to the corresponding author.

## Ethics Statement

The studies involving human participants were reviewed and approved by Ethical Committee of Fourth Hospital of Hebei Medical University. The patients/participants provided their written informed consent to participate in this study. Written informed consent was obtained from the individual(s) for the publication of any potentially identifiable images or data included in this article.

## Author Contributions

JZ and YT participated in case collection. XW writes the manuscript. GW writes the manuscript and raised idea. TL revised the manuscript and raised critical editing. All authors have read and approved the manuscript.

## Conflict of Interest

The authors declare that the research was conducted in the absence of any commercial or financial relationships that could be construed as a potential conflict of interest.

## Publisher's Note

All claims expressed in this article are solely those of the authors and do not necessarily represent those of their affiliated organizations, or those of the publisher, the editors and the reviewers. Any product that may be evaluated in this article, or claim that may be made by its manufacturer, is not guaranteed or endorsed by the publisher.
